# Solubilizing Nonpolar Substances in Polar Solvents: Strategies, Molecular Mechanisms, and Applications

**DOI:** 10.3390/ijms27104418

**Published:** 2026-05-15

**Authors:** Xiaogang Mu, Rui Wang, Shenghui Wang, Xiao Wang, Yue Zhang

**Affiliations:** Zhijian Laboratory, Rocket Force University of Engineering, Xi’an 710025, China; violet_wr1994@163.com (R.W.);

**Keywords:** solubilization, hydrophobic effect, surface and interface engineering, surfactants, green chemistry

## Abstract

Efficient solubilization of nonpolar substances in polar solvents represents a fundamental challenge in environmental remediation, green chemistry, and separation processes. This limitation stems from the hydrophobic effect, which creates thermodynamic barriers, resulting in low intrinsic solubility and strong phase separation. This review examines the thermodynamic basis of solubilization, focusing on free-energy changes and molecular interaction mechanisms. It discusses various strategies, including surface and interface engineering, host–guest inclusion, solvent engineering, and nanostructure encapsulation, along with their practical applications. Future research directions include smart responsive materials, green solvent design theories, and precise construction of solubilization systems through multi-scale simulations.

## 1. Introduction

Solubilization is the process of dispersing poorly soluble nonpolar substances into polar solvents using physical or chemical methods. This creates stable systems suitable for various applications. Common nonpolar substances include polycyclic aromatic hydrocarbons, petroleum hydrocarbons, hydrophobic industrial chemicals, and volatile organic compounds (VOC). The technology serves a key role in environmental remediation, green chemistry, industrial cleaning, and functional material synthesis. It enables efficient transport and utilization of nonpolar substances across multiple fields.

Understanding solubilization mechanisms offers both theoretical and practical benefits. The process involves fundamental phenomena like hydrophobic effects, entropy-driven assembly, and intermolecular interactions. Studying these mechanisms advances our knowledge of interface thermodynamics and solution chemistry. From an application perspective, conventional methods often require extreme conditions or large solvent volumes, giving rise to substantial energy expenses and ecological issues. Molecular-level solubilization strategies can reduce energy consumption and support green chemistry principles. This approach provides new solutions for mass transfer limitations in environmental and industrial processes.

Still, significant challenges remain in solubilizing nonpolar substances. The hydrophobic effect creates substantial free-energy barriers, resulting in low solubility and phase separation. Systems also face kinetic instability, selectivity issues, and difficulties in media regeneration. Current research focuses on achieving stable, high-capacity solubilization while developing scalable processes.

This review examines recent advances in solubilizing nonpolar substances. It analyzes four main strategies: surface engineering, host–guest systems, solvent engineering, and nanostructure encapsulation. The discussion covers thermodynamic principles, molecular mechanisms, and current limitations. Finally, it explores emerging directions like smart materials, green solvent design, and computational modeling to guide future development of efficient solubilization technologies.

## 2. Core Strategies for Solubilization

Based on the scale levels of the interaction between solubilizing media and nonpolar substances and the differences in the dominant thermodynamic driving forces, solubilizing strategies are classified into four major categories, namely surface and interface engineering, host–guest inclusion, solvent engineering, and nanostructure encapsulation, which together form a multi-scale solubilizing technology system. The following mainly discusses the three core application fields of environmental remediation, green chemistry, and chemical separation.

### 2.1. Environmental Remediation

In the field of environmental remediation, the core solution-increasing strategy is surface and interface regulation, supplemented by advanced technical means such as nanostructure encapsulation. It aims to address the issues of low solubility, poor mobility, and low degradability associated with hydrophobic refractory organic contaminants in soil and groundwater. Typical examples include polycyclic aromatic hydrocarbons, petroleum hydrocarbons, and dense non-aqueous phase liquids, while the risk of secondary pollution is also avoided.

In this strategy, by introducing surfactant molecules into the system and arranging them directionally at the interface between pollutants and the aqueous phase, the interfacial tension is significantly reduced, thereby altering the wettability and migration ability of pollutants in porous media [1]. Once the surfactant concentration rises above the critical micelle concentration, their hydrophobic tails will assemble inward to form micelles. These micelles can encapsulate hydrophobic contaminants inside, directly boosting their apparent aqueous solubility and thereby enabling the transfer of pollutants from solid or liquid sources into the aqueous phase.

#### 2.1.1. Environmental Remediation with Interface Engineering Strategy

**Figure 1:**
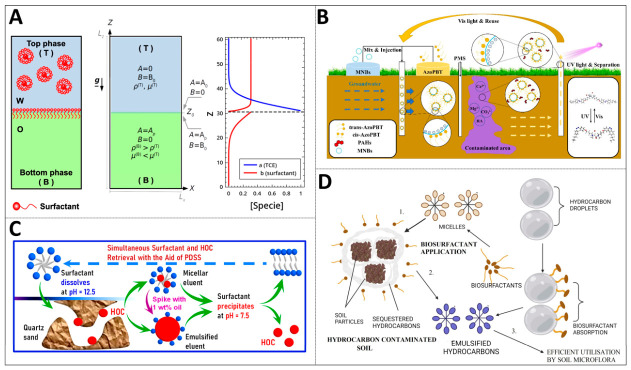
Interface engineering for nonpolar pollutant solubilization in environmental remediation: (**A**) biosurfactant-induced hydrodynamic instability enhances DNAPL solubilization (reprinted from ref. [3]; (**B**) microbial surfactant-mediated soil pollutant solubilization and remediation (reprinted with permission from ref. [10]. © 2023 Elsevier B.V. All rights reserved); (**C**) pH-triggered surfactant solubilization-recovery cycle for secondary pollution control (reprinted with permission from ref. [9]. © 2023 Elsevier B.V. All rights reserved); (**D**) photosensitive surfactant-micro–nano-bubble synergy enables cyclic PAH solubilization (reprinted with permission from ref. [8]. © 2023 Elsevier GmbH).

Surfactant-enhanced solubilization technology is a typical representative of surface and interface regulation. It has been found that certain nonionic surfactants, such as ethoxylated alcohols, not only solubilize through micellar bonding but also exhibit complex phase–behavior coupling with pollutants. When Synperonic series surfactants come into contact with dense non-aqueous phase liquids (DNAPLs) such as trichloroethylene, they can cause hydrodynamic instability at the interface, forming upward finger-like surges, as shown in Figure 1A. This convective process, driven by both local density inversion and the Marangoni effect, is dozens of times faster than the mass transfer rate of pure molecular diffusion, thereby greatly accelerating the interphase transfer of pollutants. The combination of cationic surfactants and negatively charged micro–nano-bubbles can produce a synergistic effect. Surfactants attached to the surface of microbubbles undergo alterations in their interfacial arrangement as a result of electrostatic interactions. This leads to a reduction in both the critical micelle concentration and the equilibrium surface tension of the surfactants. This, in turn, enhances the solubilization efficiency of pollutants and promotes their desorption from the contaminated medium [2,3,4,5].

The adoption of green alternative products is an important development direction for the regulation of environmental remediation interfaces. Microbial biosurfactants such as rhamnolipids and surfactants have garnered considerable interest owing to their structural diversity, low toxicity and good biodegradability. They can not only solubilize petroleum hydrocarbons through micelles, but also complex with heavy metal ions in the soil to form metal-and-biosurfactant complexes, as shown in Figure 1B, thereby achieving the synergistic removal of heavy metals and hydrocarbon pollutants [6,7,8]. The pH-responsive amphoteric surfactant 3, sodium dodecylaminopropane sulfonate, presents an intelligent recovery solution. Under alkaline conditions, this molecule exists in the form of a water-soluble salt to exert solution-increasing effects. After acidification to neutral, it transforms into an insoluble internal salt precipitate in both oil and water. Through simple pH adjustment and filtration operations, it can achieve the synchronous separation and recovery of surfactants and solubilized pollutants, avoiding the secondary pollution load caused by the disposal of flushing solutions, as illustrated in Figure 1C [9].

Photosensitive Gemini surfactants combine photoresponsiveness with high surface activity. For instance, azobenzene-type Gemini surfactants transform from *trans*-isomers to *cis*-isomers under ultraviolet light irradiation, with weakened hydrophobicity leading to micelle dissociation and the release of solubilized polycyclic aromatic hydrocarbons. Visible light irradiation can also restore its solution-increasing capacity. Combined with the synergistic effect of micro–nano-bubbles, as shown in Figure 1D, it provides a recyclable surface and interface enhancement solution for the in situ treatment of PAH-contaminated groundwater [3,10]. These strategies collectively reflect the trend of evolution in environmental remediation from simple solubilization to intelligent, efficient and recyclable precise surface and interface regulation.

#### 2.1.2. Environmental Remediation with Nanostructured Encapsulation Strategy

By means of the microemulsion, which is a nano-dispersion system, non-aqueous liquid pollutants are dispersed into nanoscale droplets, significantly increasing the oil–water interface area and enhancing the degradation efficiency of pollutants by microorganisms. The repair and recovery efficiency of microemulsions formulated with nonionic and anionic surfactants is superior to that of cationic surfactants [11].

**Figure 2:**
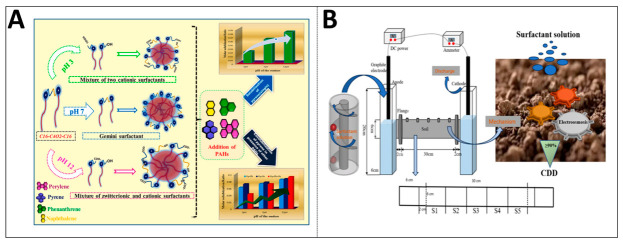
Nanostructured encapsulation of hydrophobic organic pollutants: micellization and electroosmotic synergism. (**A**) pH-responsive micellar encapsulation of PAHs (reprinted with permission from ref. [12]. © 2023 Elsevier Ltd. All rights reserved); (**B**) electroosmotic flow-driven nanoscale desorption (reprinted with permission from ref. [14]. Copyright © 2024, The Author(s), under exclusive license to Springer Nature Switzerland AG).

By regulating the pH value of the in situ mixed micelles of ester-functionalized twin surfactants, the solubilization and co-solubilization effects of different hydrophobic polycyclic aromatic hydrocarbons can be controlled. As shown in Figure 2A, this type of surfactant undergoes ester bond hydrolysis under non-neutral pH conditions, generating single-tail surfactant components with different charge characteristics. It further forms in situ mixed micelles with distinct characteristics. This PH-triggered polymorphic transformation enables it to exhibit adjustable solubilization capabilities and synergistic or competitive co-solubilization behaviors for polycyclic aromatic hydrocarbons (PAHs) such as naphthalene, phenanthrene, pyrene, and perylene [12]. Nano-encapsulated transition metal nanoparticles can enhance the catalytic activity, stability and selectivity of advanced oxidation processes, and efficiently degrade organic pollutants [13].

The high reactivity inherent to nanostructures can effectively strengthen the desorption and migration performance of semivolatile organic contaminants present in soil. This in turn elevates the bioavailability of these target pollutants. As shown in Figure 2B, surfactants play a significant role in the electrodynamic remediation process. They facilitate the transport of pollutants, including cyclododecane, into the aqueous phase by lowering interfacial tension and strengthening electroosmosis. Among them, the anionic surfactant SDBS exhibits superior removal efficiency compared to the nonionic surfactant AEO due to its ability to provide higher current and cumulative electroosmotic flow [14]. Cellulose nanocrystal-stabilized Pickering emulsions can also promote the solubilization and desorption of petroleum hydrocarbons through their nanostructural properties [6]. The nanoscale interaction form of the cationic surfactant DTAB combined with nanobubbles can significantly enhance the solubilization effect of phenanthyl [5].

### 2.2. Green Chemistry

The core solubilization strategies within green chemistry center on solvent engineering and host–guest inclusion. Solvent engineering aims to replace traditional toxic organic solvents by developing or optimizing the solvents themselves and to construct environmentally friendly reaction systems. The main inclusion strategy optimizes the water-phase reaction efficiency by encapsulating the reactants within a specific structure. These two approaches comply with the fundamental tenet of green chemistry, which aims to reduce the utilization and production of toxic substances as much as possible [15,16].

#### 2.2.1. Green Chemistry with Solvent Engineering Strategy

The development of solvent engineering is particularly prominent. Deep eutectic solvents and natural deep eutectic solvents, as representative green solvents, have garnered widespread attention owing to their low toxicity, biodegradability and recyclability [17,18,19,20,21]. These solvents have found successful applications in various fields, such as biomass fractionation and high-value utilization, solubilization of poorly soluble drugs, and green organic synthesis. They are also employed in the ultrasound-enhanced extraction of bioactive substances, greenhouse gas capture, and metal recovery from electronic waste [22,23,24,25], in some application scenarios, they can also serve as dual carriers of solvents and catalysts, thereby achieving high-yield synthesis.

**Figure 3:**
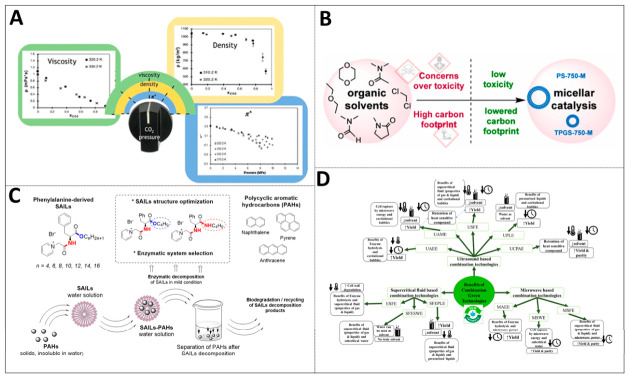
Overview of multi-domain applications and synergistic strategies of green solvents: (**A**) applications of deep eutectic solvents in biomass fractionation and green synthesis (reprinted with permission from ref. [27]. Copyright © 2019, American Chemical Society); (**B**) supercritical CO_2_-regulated solvent systems for clean manufacturing and separation (reprinted with permission from ref. [29]. Copyright © 2024, American Chemical Society); (**C**) “on-water” organic synthesis and micellar catalysis strategies (reprinted from ref. [33]; (**D**) solubilization of hydrophobic pollutants by biodegradable surface-active ionic liquids, reprinted with permission from ref. [32]. © 2023 Elsevier B.V. All rights reserved).

Constructing a two-phase system of supercritical carbon dioxide and green solvents is another important direction in solvent engineering. By precisely regulating the pressure of carbon dioxide, the solvation and mass transfer properties of the system can be significantly altered. Existing investigations demonstrate that with increasing CO_2_ pressure, the polarity parameters of the solvent decrease markedly and can even become negative. Mass-transfer properties such as density and viscosity also drop substantially under these conditions. As an illustration, in certain straight-chain organic carbonate systems, the density may be lowered to half that of neat solvents, with viscosity declining by as much as 90% [26]. This precise regulation ability makes it highly applicable in the manufacturing of clean chemicals. By regulating the viscosity, density and π* polarity parameters of bio-based solvents through CO_2_ expansion, the viscosity can be reduced by up to 90%, the density can be decreased to 50%, and the polarity can be continuously adjusted within a wide range, thereby achieving efficient mass transfer and solvent recovery. Furthermore, the utilization of green solvents in polymer solar cell processing and the design of pure-water mobile phases for chromatography has effectively reduced the production of organic waste, as indicated by Figure 3A [27,28,29].

Waterborne organic synthesis can be achieved by using water-based solvents in combination with water-soluble growth aids or other solubilizers. The micelle catalytic strategy utilizes the micelles formed by surfactants as nanoreactors to provide a suitable microenvironment for hydrophobic reactants, thereby effectively enhancing the reaction rate and selectivity. In industrial practice, micellar catalysis has been successfully applied to the synthesis of various drug intermediates, which can significantly reduce the usage of precious metal catalysts and toxic solvents, thereby improving the quality, strength and cost of the process (see Figure 3B) [15,30]. Smart solvents, including green ionic liquids and deep eutectic solvents, likewise exhibit outstanding solubilization performance and environmental compatibility, as shown in Figure 3D [31,32].

The surfactant ionic liquid derived from phenylalanine can effectively solubilize polycyclic aromatic hydrocarbons. Its solubilization capacity increases with the growth of alkyl chains and can eventually reach a level comparable to or even better than that of traditional cationic surfactants. this type of surface-active ionic liquid also has the advantage of breaking ester or amide bonds through enzymatic hydrolysis under mild conditions, thus enabling in situ separation and recycling of the system, as shown in Figure 3C. This reflects the idea of pursuing sustainability from the source of molecular design [33].

#### 2.2.2. Green Chemistry with Host–Guest Inclusion Strategy

Hydrophobic substrates are encapsulated via hydrophobic interactions and other non-covalent forces, including hydrogen bonding, using renewable and biodegradable main molecules such as cyclodextrin [34,35]. The inclusion strategy constructs a hydrophobic microenvironment similar to enzyme active pockets in the aqueous phase, effectively achieving substrate solubilization and activation of reaction sites. This approach avoids the employment of conventional organic solvents and greatly lowers the release of volatile organic chemicals. Moreover, it improves the atomic economy of the reaction by elevating the local substrate concentration as well as the reaction selectivity [16].

**Figure 4:**
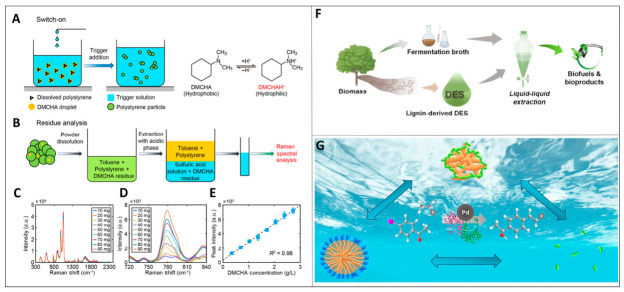
Host–guest inclusion and green solvent systems for promoting organic synthesis in aqueous phase: (**A**) Schematic of phase switching and dissolution process for switchable hydrophilicity solvents (reprinted from ref. [36]); (**B**) size distribution and morphological characterization of polystyrene microparticles (reprinted from ref. [36]); (**C**) effect of trigger addition time on solvent residue (reprinted from ref. [36]); (**D**) effect of trigger concentration on solvent residue (reprinted from ref. [36]); (**E**) effect of organic phase composition on solvent residue and water entrapment mechanics (reprinted from ref. [36]); (**F**) molecular structures of lignin-derived deep eutectic solvents (reprinted with permission from ref. [37]. © 2022 Elsevier Ltd. All rights reserved); (**G**) application of surfactant-free microemulsions and binary mixtures in cascade reactions (reprinted with permission from ref. [38]. © 2023 Elsevier B.V. All rights reserved).

Switchable hydrophilic solvents (SHS), with their unique characteristic of reversible switching between hydrophobicity and hydrophilicity, offer an innovative path for green separation technology. Tertiary amine compounds represented by N,N-dimethylcyclohexylamine undergo protonation when in contact with CO_2_ or acidic triggers, transforming from a hydrophobic state to a hydrophilic state. This process is completely reversible. By removing CO_2_ or adjusting the pH, the hydrophobic form can be restored, achieving efficient recovery and recycling of the solvent. Research shows that the shift in Raman spectral intensity with concentration variation can monitor in real time the quantitative changes in residues during the solvent recovery process, providing direct experimental evidence for this reversibility. During the preparation of polymer particles, SHS effectively controls the solvent residue through droplet emulsification and phase switching mechanisms, optimizing the sustainability of the chemical process, as presented in Figure 4A–E [36]. As a newly developed eco-friendly solvent, deep eutectic solvent (DES) is formed through hydrogen-bonding interactions between hydrogen bond donors and acceptors, with tunable physicochemical properties. Notably, hydrophobic deep eutectic solvents prepared from lignin-derived compounds, including menthol, thymol and 2,6-dimethoxyphenol, show low viscosity (16–40 mPa·s) and superior extraction performance. Molecular dynamics simulations indicate that DES possesses an intricate hydrogen-bonding network inside, which can effectively solubilize organic molecules, including acetone and n-butanol. Modulating the molar ratio of the individual components enables optimization of the distribution coefficient and selectivity toward target substances. When integrated with host–guest encapsulation, this strategy can synergistically facilitate green organic synthesis and the high-value utilization of biomass (see Figure 4F) [22,37]. In aqueous catalytic reactions, the inclusion of the main molecule or the construction of surface-free microemulsions (SFMEs) can simultaneously dissolve hydrophobic substrates and protect the active conformation of enzyme catalysts. This strategy has enabled the efficient integration of transition metal catalysis (represented by Heck reactions) and biocatalysis (typified by alcohol dehydrogenase reduction reactions). By forming a micellar structure with a hydrophobic core, not only is the solubility of the substrate enhanced, but a suitable microenvironment is also provided for the cascade reaction, significantly improving the efficiency of aqueous phase catalytic reactions and promoting the development of sustainable chemical synthesis, as depicted in Figure 4G [38].

### 2.3. Chemical Separation

In the field of chemical separation, the main package strategy can be adopted. Relying on the molecular specific recognition ability, energy-saving separation processes can be developed. Meanwhile, surface and interface engineering strategies can be incorporated as a supplementary interface control technique. This helps overcome the drawbacks of high energy consumption, heavy pollution and low selectivity in conventional separation processes, while enhancing both separation efficiency and process sustainability [34,39].

#### 2.3.1. Chemical Separation with Host–Guest Inclusion Strategy

**Figure 5:**
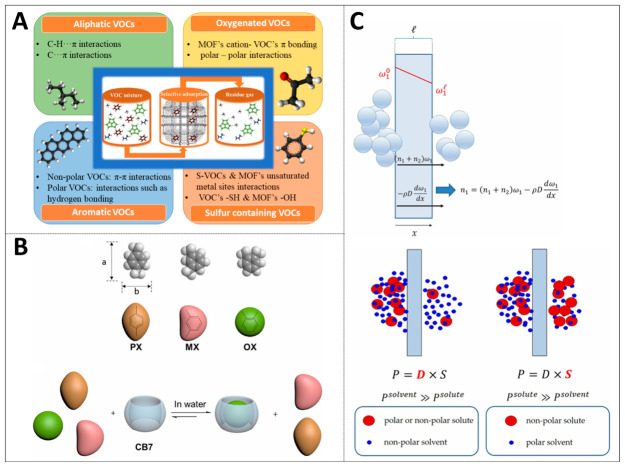
Specifically identified energy-saving separation strategies: (**A**) regulation of hydrophilicity and hydrophobicity of MOFs (reprinted with permission from ref. [41]. © 2023 Elsevier B.V. All rights reserved); (**B**) CB7 molecular shape recognition (reprinted with permission from ref. [34]. © 2020 Elsevier Inc.); (**C**) interface transfer regulation of nanofiltration membranes (reprinted from ref. [44]).

The main inclusion strategy utilizes the inclusion and adsorption capacity of cyclodextrin-based materials to remove pollutants such as pesticides, drugs, and dyes from wastewater [40]. As shown in Figure 5A, metal–organic frameworks (MOFs) feature a tunable framework and sizable specific surface area. Owing to these advantages, they have emerged as high-performance adsorbents for the capture of volatile organic compounds [41].

Specifically, aliphatic hydrocarbons bind to the pore walls of the framework through van der Waals dipole interactions, aromatic hydrocarbons mainly form π and π host–guest interactions, oxygen-containing VOCs rely on polar interactions to bind to suitable adsorption sites, and sulfur-containing VOCs tend to form strong bonds with frameworks containing unsaturated metal sites. Cucurbituron and other macrocyclic compounds exhibit unique advantages in the separation of isomers [34,42]. Cucurbituron’s aqueous solution can efficiently separate ortho-disubstituted benzene isomers through liquid–liquid extraction at normal temperature and pressure, with a selectivity exceeding 92%. This process is based on the shape matching effect. The ortho-isomers, because they have the smallest aspect ratio, have the highest fit with the spherical cavity of cucurbituron, resulting in more thermodynamic stability and slower dissociation kinetic rates, as illustrated in Figure 5B. Isothermal titration calorimetry measurements revealed much higher binding constants for o-xylene with cucurbituril compared to xylene and m-xylene. Specifically, the values were 27-fold and 21-fold higher, respectively, while the dissociation rate constants were notably lower than those of other isomers. This strategy has been effectively utilized for separating o-xylene from commercial xylene mixtures and the C8 fraction of cracked gasoline. The selectivity of industrial sample separation reaches 83%, which is significantly better than traditional crystallization or adsorption processes [34]. This type of material can also achieve the separation of traditional difficult-to-separate systems such as toluene and n-heptane [43].

Organic solvent nanofiltration membrane technology can be combined with the main inclusion strategy to achieve low-energy consumption and low-carbon footprint separation of molecules in nonpolar solvent systems, replacing traditional distillation techniques [44]. This membrane material is based on the solution–diffusion mechanism, and the related formula provides a theoretical basis for understanding how OSN membranes achieve efficient separation, and efficient separation itself is the key way to reduce energy consumption. By adjusting the free volume and chemical composition, OSN membranes can operate at room temperature without phase change, and the energy consumption is significantly lower than that of thermal separation processes, as proven in Figure 5C. The retention and permeability of solutes by membranes follow the trade-off relationship between solubility and selectivity. By designing microporous polymers or mixed matrix membranes with controllable pore sizes, it is expected to achieve efficient screening of structurally similar molecules.

#### 2.3.2. Chemical Separation with Interface Engineering Strategy

**Figure 6:**
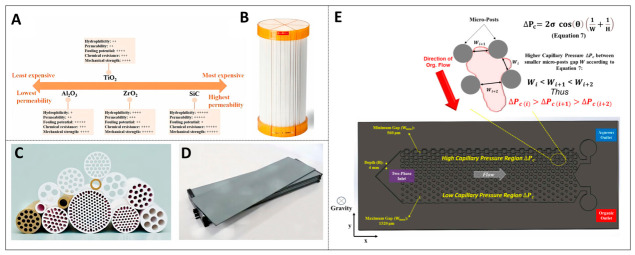
Interfacial engineering-based separation technologies: (**A**–**D**) the permeability and retention performance of OSN membranes in nonpolar solvent systems (reprinted from ref. [45]); (**E**) capillary-driven phase separation mechanism of microfluidic separator (reprinted with permission from ref. [46]. © 2024 Elsevier B.V. All rights reserved).

Interface engineering strategies provide an effective way to regulate the surface properties of organic solvent nanofiltration membrane materials. Hydrophobic functional groups—including fluorinated segments, long-chain alkyls, or siloxane moieties—can be grafted onto the membrane surface [45]. This modification greatly strengthens the attraction of the membrane matrix toward nonpolar solvents. Such chemical modification optimizes the interaction at the three-phase interface of the membrane, solvent and solute, achieving selective solubilization and permeation of specific molecules. In specific implementation, the interfacial polymerization method is adopted to introduce fluorine-containing monomers or amphiphilic multi-block oligomamines into the selective cortex, which can construct ultrathin separation layers with enriched hydrophobic channels [46]. Such membrane materials show markedly enhanced permeability toward nonpolar organic solvents like toluene and n-hexane, often by one order of magnitude or higher. At the same time, they maintain efficient rejection performance for target solutes. Coating modification on the surface of the support layer (such as spin-coated polydimethylsiloxane or perfluoropolyether) and silanization, a hydrophobic treatment of ceramic membranes, can also reduce mass transfer resistance. By precisely tuning surface wettability, micro-roughness and interfacial charge properties, these strategies improve the chemical stability and separation selectivity of the membrane in nonpolar media. This effectively lowers the energy consumption of separation and offers a high-efficiency, energy-saving technical route for industrial processes, including hydrocarbon fractionation and pharmaceutical refining, as illustrated in Figure 6A–D [47]. Multifunctional interfacial active materials (MIAMs) can enhance interface reconstruction and separation performance, and efficiently handle unconventional liquids and liquid systems.

In the field of microfluidic separation technology, additive manufacturing methods (especially binder jetting) are used to construct new high-throughput multiphase separators. The microcolumn array structure inside this device can generate precise capillary pressure gradients during the flow process [48]. According to the Young and Laplace equations, the lateral gradient change in the microcolumn gap (for example, from 560 μm to 1520 μm) will form a pressure difference on both sides of the non-wetting phase, driving the organic phase to migrate directionally to the low-pressure region, while the aqueous phase is confined to a specific flow channel due to capillary resistance. This separation mechanism, which relies on differences in surface wettability, completely abandons the dependence of conventional membrane separation on porous media. It thereby enables a continuous phase separation process that requires no membrane and is independent of density variations [49]. Such a separator can efficiently handle various flow patterns, such as sectional plug flow and dispersed droplet flow, and still maintain a phase purity of over 95% under operating conditions with a total flow rate as high as 15 mL/min.

Due to the elimination of membrane fouling risks and the advantages of a simple structure and low-cost manufacturing, this device demonstrates excellent potential for industrial scale-up and is particularly suitable for solvent extraction and biofuel separation processes that require enhanced mass transfer and rapid phase separation [50]. By taking advantage of the weak surface activity of some amphiphilic compounds, the separation and enrichment of hydrophobic organic substances from dilute aqueous solutions can be achieved at low cost and with easy operation through ultrasonic emulsion enrichment, as presented in Figure 6E.

## 3. Mechanism of Solubilization

The solubilization mechanism plays a crucial role in environmental remediation, green chemical processes, and chemical separation fields. It disperses and stabilizes nonpolar substances with extremely low intrinsic solubility in polar solvents through physical or chemical means, forming thermodynamically or kinetically stable homogeneous or heterogeneous dispersion systems, thereby enhancing the migration, degradation, or separation efficiency of insoluble pollutants.

### 3.1. Intermolecular Interaction

#### 3.1.1. Hydrophobic Effect

**Figure 7:**
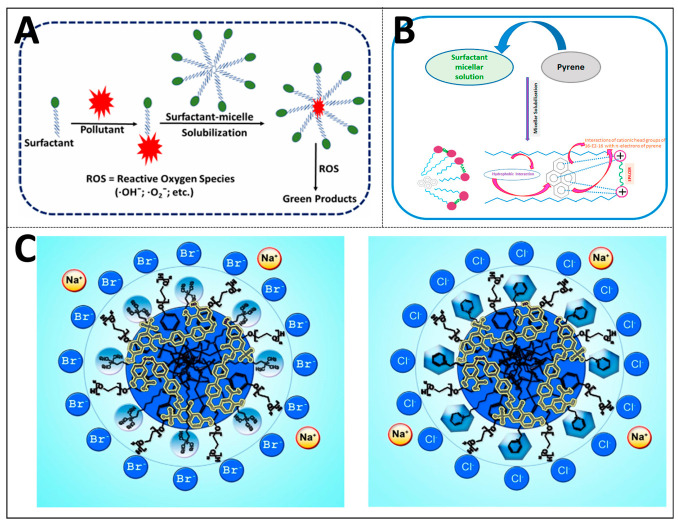
Molecular structures and interactions of surfactants and pollutants: (**A**) structures of cationic surfactants and direct dye (reprinted from ref. [51]); (**B**) structures of Gemini surfactants and polycyclic aromatic hydrocarbons (reprinted with permission from ref. [53]. © 2020 Elsevier B.V. All rights reserved); (**C**) structures of cationic and nonionic mixed surfactant systems and dye (reprinted with permission from ref. [52]. © 2019 Published by Elsevier B.V).

Intermolecular interactions are the foundation that supports this process. Hydrophobicity, as the core driving force, can prompt nonpolar substances such as polycyclic aromatic hydrocarbons, chlorinated hydrocarbons, and petroleum hydrocarbons to escape from the aqueous phase and then enter the core of surfactant micelles or cyclodextrin-encapsulated cavities [51,52,53]. Amphiphilic surfactant molecules in aqueous solutions will spontaneously aggregate into micelles, within which a hydrophobic microenvironment is constructed to effectively accommodate these pollutants, as shown in Figure 7A–C [1,51,52,54,55,56]. The nature of this effect is a remarkable rise in the entropy of the aqueous system. When the hydrophobic surface is shielded, the ordered water molecules originally arranged around hydrophobic substances are released back into the bulk phase. These water molecules return to a state of free rotation, enabling the entire solubilization process to occur spontaneously. Structurally unique dual-quaternary ammonium salt Gemini surfactants can efficiently solubilize typical polycyclic aromatic contaminants, including naphthalene, anthracene and pyrene, via this mechanism [51]. When dealing with membrane proteins, it is also necessary to overcome the hydrophobic effect. The specific approach is to use amphiphilic detergent molecules to coat the hydrophobic transmembrane regions of the proteins, forming water-soluble protein–detergent complexes.

#### 3.1.2. Van der Waals and π-π Stacking Forces

Beyond hydrophobic interactions, van der Waals forces and π–π stacking also exert equally crucial roles during the solubilization of aromatic compounds. Nuclear magnetic resonance studies have shown that the ring current generated by the π electrons of aromatic rings can induce chemical shifts in adjacent surfactant molecules. This interaction between π-electrons and polar head groups governs the specific location of PAHs within micellar structures. Owing to the weak binding between π-electrons and negatively charged polar heads, the solubilization capacity of PAHs in anionic surfactant micelles is generally lower than that in cationic ones. Anthracene with low hydrophobicity tends to be distributed in the micellar fence layer and shell area, while pyrene with stronger hydrophobicity is more likely to penetrate into the hydrophobic core. Different surfactant systems have selectivity in solubilizing pollutants. Sodium dodecyl sulfate has a better removal effect on aliphatic hydrocarbons, while saponins and other substances have more advantages in treating aromatic hydrocarbons. The mixed system constructed by quaternary ammonium salt surfactants and conventional surfactants can alter the micelle structure and internal hydrophobic microenvironment. Combined with van der Waals interactions, π–π stacking forces and intermolecular synergy, it significantly enhances the solubilization capacity for substances such as polycyclic aromatic hydrocarbons [53,55,56]. In the practical remediation of soil contaminated by polycyclic aromatic hydrocarbons, iron–hydroxylamine carriers have been employed. Through the synergistic effects of cations, π-interactions and chelation, these carriers enable the simultaneous removal of heavy metal ions and persistent aromatic pollutants [57].

#### 3.1.3. Subject–Object Interaction

The solubilization pathway of cyclodextrin, as another important type of solubilizer, depends on host–guest interactions [58,59,60]. This type of molecule has a special cavity structure that is hydrophilic on the outside and hydrophobic on the inside, which can enclose hydrophobic guest molecules within it to enhance the solubility of the aqueous phase [59,60,61,62]. The generation and structural stability of inclusion complexes are governed by a variety of driving forces. These include the liberation of high-energy water molecules confined within the host cavity, hydrophobic interactions, van der Waals forces, and hydrogen bonding. The size matching degree and polarity complementarity of the guest molecules directly determine the inclusion efficiency. Because inclusion is essentially a spatial adaptation process between the subject cavity and the object molecule. Hydrophobic objects can replace the water molecules with strong polarity and unfavorable energy in the cavity, thereby providing a favorable net energetic driving force for the insertion of guest molecules into the cyclodextrin cavity. Derivatized cyclodextrins exhibit superior solubilization performance compared to natural cyclodextrins due to their stronger water solubility. Cyclodextrin not only enhances the apparent water solubility of pollutants but also forms a ternary complex of pollutants, cyclodextrin and iron with iron ions, thereby guiding hydroxyl radicals to directly act on pollutants during the subsequent advanced oxidation and degradation process and improving the treatment efficiency. β-Cyclodextrins and their modified derivatives have been demonstrated to improve the bioavailability of petroleum hydrocarbons in polluted soil, thus facilitating the phytoremediation process. Cyclodextrin derivatives can further construct ternary complexes of pollutants, cyclodextrins and polymers, thereby enhancing the dissolution and removal effect of hydrophobic organic pollutants in the soil [59,63].

#### 3.1.4. Hydrogen Bonds Interact with Electrostatic Forces

Hydrogen bonds and electrostatic interactions play a key regulatory role in various solubilization systems. Owing to their low vapor pressure and excellent thermal stability, ionic liquids have been recognized as environmentally benign solvents [64,65,66,67]. Their cations and anions can combine with solute molecules through hydrogen bond acceptor–donor interaction and electrostatic interaction, significantly altering the distribution behavior of solutes in the liquid phase [66,68,69]. Computational chemistry methods based on the COSMO-RS theory have been used to screen suitable ionic liquids. Combined with quantum chemical calculations, the hydrogen bond network and electrostatic interaction mode between ionic liquids and solute molecules can be analyzed in depth, thereby clarifying the microscopic mechanism by which they promote separation. Co-solvent blends, including ethanol–water and ethyl lactate–water mixtures, exhibit excellent solubilizing performance. These systems can greatly boost the dissolution of hydrophobic organic compounds via intermolecular interactions such as hydrogen bonding and van der Waals forces between solvent molecules and polycyclic aromatic hydrocarbons [70].

**Figure 8:**
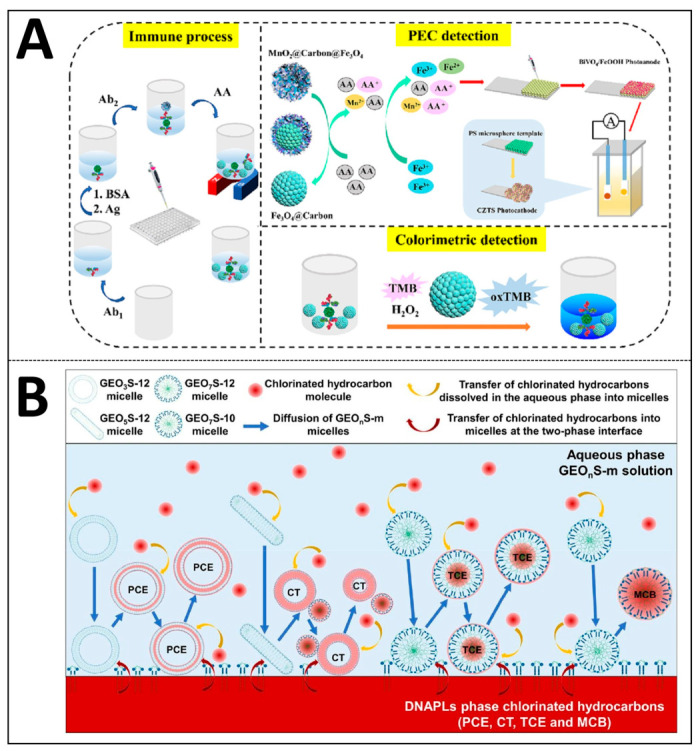
Nanostructure and solubilization-sensing mechanisms: (**A**) morphological characteristics of functionalized nanocomposites (reprinted with permission from ref. [68]. © 2023 Elsevier B.V. All rights reserved); (**B**) structures and solubilization sites of surfactant micelles (reprinted with permission from ref. [69]. © 2023 Elsevier B.V. All rights reserved).

Functionalized nanocarriers can precisely control the interaction intensity with target molecules through surface modification. Taking the manganese dioxide-coated iron (iii) oxide@carbon nanosphere complex (Fe_3_O_4_@Carbon@MnO_2_) as an example, the core–shell structure forms a carbon layer on the Fe_3_O_4_ surface through dopamine autopolymerization and high-temperature carbonization, and then the hydrothermal growth of MnO_2_ nanosheets constructs a sandwich structure. After the surface is functionalized with amino silane (APTES) and glutaraldehyde, it can covalently link biometric molecules (such as antibody Ab2). During the solubilization and separation process, the outer MnO_2_ shell can be selectively etched and shed by ascorbic acid, exposing the Fe_3_O_4_@Carbon core with peroxidase-like activity. The exposed surface captures target molecules through electrostatic interactions and hydrogen bonds, while providing a catalytic reaction interface, as shown in Figure 8A [71].

The solubilization ability of surfactant micelles is significantly affected by their aggregation morphology and surface charge distribution. Anionic and nonionic Gemini surfactants (such as the Dungeons-m series) change their aggregate morphology from vesicles to rod-like micelles with the increasing ethoxy (EO) units, and eventually form spherical micelles. Micelles of different forms provide differentiated solubilization microenvironments: nonpolar chlorinated hydrocarbons (such as tetrachloroethylene and carbon tetrachloride) are mainly solubilized in the hydrophobic core. Polar chlorinated hydrocarbons (such as trichloroethylene and chlorobenzene) are mainly distributed in the palisade layer. Particularly importantly, halogen atoms in chlorinated hydrocarbon molecules can form halogen bonds (R-Cl···O) with oxygen atoms in the EO units of surfactants. This directional electrostatic interaction and hydrophobic effect work in synergy to regulate the solubilization process, and its existence can be confirmed by the displacement of the characteristic peak of the C-Cl bond in the infrared spectrum [72], Figure 8B presents a model of the micellar structure of an anion–non-ion triblock surfactant for the solubilization of chlorinated hydrocarbons, illustrating the distribution sites of different polar chlorinated hydrocarbons within the micelles. Mixed surfactant systems, for instance, combinations of cationic and nonionic types, can further improve solubilization performance toward specific pollutants like azo dyes. This is achieved by regulating the surface charge distribution of micelles and the structural characteristics of hydrophobic microdomains [55].

### 3.2. Thermodynamics and Dynamics

#### 3.2.1. Thermodynamic Model

**Figure 9:**
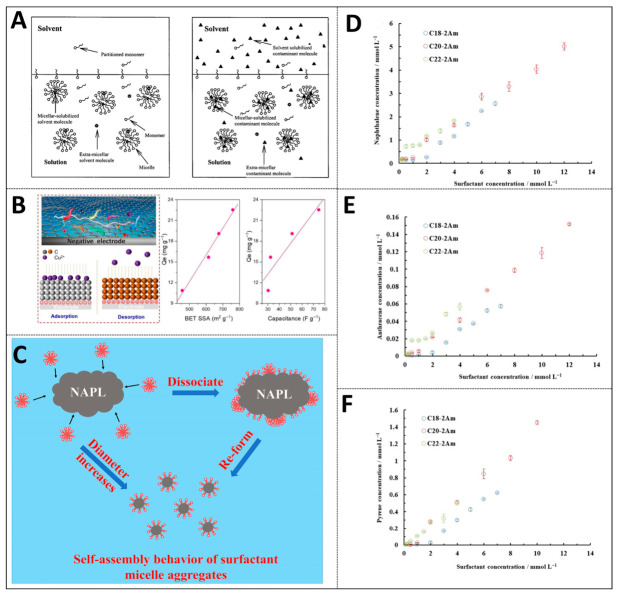
Thermodynamic and kinetic mechanisms of surfactant solubilization and pollutant remediation: (**A**) solvent extraction of micellar-solubilized contaminants and anionic surfactants: thermodynamic and kinetic analysis (reprinted with permission from ref. [70]. Copyright © 2001, American Chemical Society); (**B**) electro-adsorption/desorption of pollutants on carbon monoliths: kinetic process (reprinted with permission from ref. [73]. © 2023 Elsevier B.V. All rights reserved); (**C**) self-assembly behavior of surfactant micelles and pollutant phase transfer: thermodynamic mechanism (reprinted with permission from ref. [2]. © 2023 Elsevier B.V. All rights reserved); (**D**–**F**) solubilization of PAHs by supralong-chain surfactants: thermodynamic and kinetic study (reprinted with permission from ref. [47]. © 2024 Elsevier B.V. All rights are reserved, including those for text and data mining, AI training, and similar technologies).

Phase solubility graphs can quantitatively characterize the solubilization capacity of solubilizers (such as surfactants) for insoluble organic pollutants [52]. Once the surfactant concentration rises above the critical micelle concentration (CMC), micelles begin to form in large quantities. Beyond this point, the solubility of the solute shows a linear increase as the surfactant concentration continues to grow [53,55]. The molar solubilization ratio (MSR) could be determined through the slope of this linear segment. This parameter directly quantifies the solubilizing ability of a unit concentration of surfactant toward specific pollutants. For poorly soluble organic compounds like polycyclic aromatic hydrocarbons, the MSR rises noticeably as the alkyl chain length of the surfactant increases. For instance, the MSR of the ultra-long-chain surfactant C22-2Am against naphthalene can reach 0.48, which is much higher than the 0.27 of the conventional C16-Am. CMC is the key threshold. When the surfactant concentration reaches this value, the molecules will spontaneously aggregate into micelles. Micelle formation is primarily driven by the hydrophobic effect, an entropy-dominated process where ordered water molecules surrounding the surfactant tails are released into the bulk phase. The thermodynamic tendency of this process can be evaluated using the Gibbs free-energy change (ΔG^0^). A more negative ΔG^0^ value indicates a stronger driving force for pollutants to migrate from the aqueous phase into the micellar phase. Due to their larger hydrophobic core and barrier layer regions, the ΔG^0^ of the solubilization process of very long-chain surfactants can be as low as −35 kJ/mol, providing more favorable thermodynamic conditions for solubilization (see Figure 9D–F) [51].

The micelle and water distribution coefficient (K_m_) is a key parameter for predicting the migration potential of pollutants (such as PAHs) in micelle-enhanced remediation (such as surface-enhanced groundwater remediation, SEAR). This coefficient reflects the distribution balance of pollutants between the micelle phase and the aqueous phase, and has a quantitative relationship with MSR. It can be calculated by the formula:

(1) $$K_{m}=\frac{MSR}{1+MSR}\times \frac{55.556}{C_{w}}$$

where C_w_ is the solubility of pollutants in water. Equation (1) provides the quantitative relationship between the micelle–water distribution coefficient and the molar solubilization ratio. Solvent extraction can be applied to separate micelle-solubilized contaminants from anionic surfactants. Its effectiveness is influenced by several factors, including surfactant concentration, solution ionic strength, solvent solubilizing capability, and the volume ratio between solvent and aqueous solution. Specifically, the equivalent alkyl carbon number (EACN) of the extraction solvent needs to be much higher than the EACN value of the target pollutant to reduce the degree to which the solvent is solubilized by micelles, thereby avoiding a decrease in the efficiency of pollutant removal. However, solvents with an excessively high EACN may reduce the mass transfer efficiency due to a decrease in the number of molecules per unit volume caused by an increase in molecular weight. Therefore, the balance between solvent solubilization and molecular weight needs to be comprehensively considered. In addition, increasing salinity can boost pollutant solubilization by lowering the CMC and driving micelles to transform from spherical to rod-like structures, as shown in Figure 9A. However, this also intensifies the solubilization of the extractant within micelles. Consequently, the distribution coefficient *K_m_* between the organic phase and the aqueous phase declines, which in turn lowers the overall extraction performance [2,73].

#### 3.2.2. Dynamic Process

The solubilization rate directly affects the leaching remediation cycle of contaminated soil [59]. This process is not only governed by equilibrium thermodynamics. Its kinetic rate also relies heavily on the assembly characteristics of surfactant micelles and the dynamic evolution of aggregate size. It is shown that during the solubilization process, micelles undergo self-assembly behavior of dissociation and re-aggregation. Surfactant monomers attach to the NAPL-and-water interface and carry pollutant molecules to re-aggregate and form larger aggregates, thereby accelerating phase transfer. For anionic/amphoteric hybrid surfactant systems (such as SDBS/BS-12), owing to the generation of densely packed mixed micelles via electrostatic attraction, the phase transfer rate is 7.4–33.8% higher than that of single surfactants. This is attributed to the combined effect of the reduction in CMC in the hybrid system and the increase in aggregate size, which leads to an increasing total surface area, as depicted in Figure 9C [2]. For instance, in the research on the removal of various chlorinated hydrocarbons from groundwater by in situ microemulsions, the formation and solvation behavior of microemulsions and their influencing factors (such as hydrogeochemical conditions) are crucial [74]. Silicone oil emulsions have been investigated for improving the recovery of chlorinated solvents, including trichloroethylene and perchloroethylene. Their solubilization performance and kinetic rate were assessed through both batch tests and column experiments [75].

The degradation and release kinetics of carrier materials are equally crucial in environmental remediation. The adsorption process of the mesoporous carbon monolith (MCM) under electric field assistance conforms to the pseudo-second-order kinetic model, which suggests that chemisorption serves as the governing mechanism. Applying voltage can significantly shorten the time to reach adsorption equilibrium. Under a voltage of 0.5 V, the adsorption capacity for Cu^2+^ can reach 2.56 wt. % within 120 min, which is much higher than the equilibrium capacity under the condition of no electric field. Furthermore, the adsorption capacity exhibits a positive linear relationship with the specific surface area as well as specific capacitance of the material. The in situ regeneration process achieves desorption through reverse voltage. The desorption kinetics is affected by the electrolyte concentration and flow rate. As illustrated in Figure 9B, efficient electro-desorption can be achieved using a 10 g/L sodium sulfate solution at 85 ± 5 °C and a flow rate of 20 mL/min, enabling the MCM to retain a stable adsorption capacity of 2.71 wt. % after eight cycles and exhibiting outstanding kinetic reversibility [76]. In bioremediation, the addition of β-cyclodextrin can enhance the bioavailability of petroleum hydrocarbons, thereby accelerating their degradation process [63].

## 4. Challenges and Prospects

Solubility-based technologies have broad application prospects in environmental remediation, green chemical processes, and chemical separation fields. However, their development still faces many technical challenges. Meanwhile, future development trends will focus on intelligent response systems, multi-strategy synergy, computational simulation-driven design, and enhanced green sustainability.

### 4.1. Existing Limitations

Firstly, several conventional solubilizers possess relatively high ecological toxicity and may pose potential ecological risks and health hazards to the surrounding environment and human beings. Traditional organic solvents, including *N*-methyl-2-pyrrolidone (NMP), *N*,*N*-dimethylformamide (DMF), and *N*,*N*-dimethylacetamide (DMAC), are being phased out and substituted with eco-friendly solvents on account of their inherent toxicity and adverse environmental effects [77,78,79,80]. In environmental remediation, chlorinated hydrocarbon pollutants usually exist in aquifers in the form of complex mixtures, and their solubilization behavior is influenced by the formation of microemulsions and selective solubilization mechanisms [74].

Secondly, the carrier materials used for solubilization also face stability challenges in practical applications. Micelles and microemulsions may suffer structural damage under extreme pH, high temperature or high ionic strength conditions, leading to a decline in solubilizing efficiency or even failure. This instability limits its wide application in large-scale industrial production [74].

**Figure 10:**
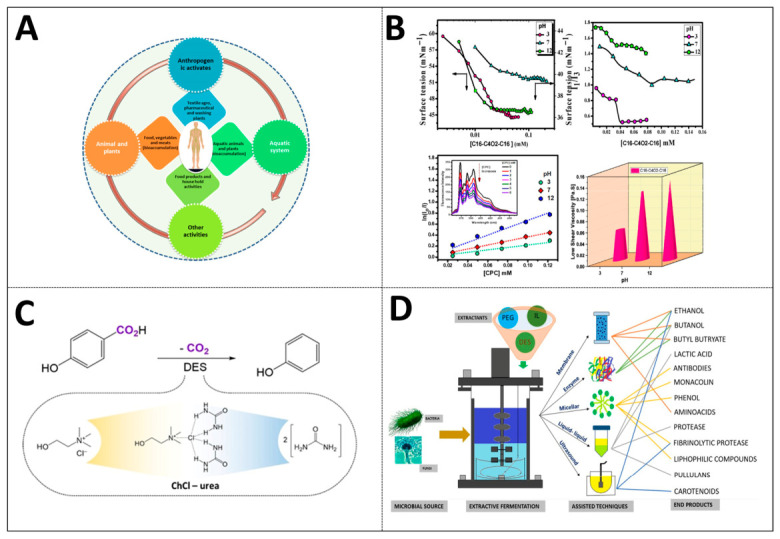
Application barriers and performance limitations of solubilization technology in environmental remediation green chemistry and chemical separation fields: (**A**) application of green solvents in sample preparation and their green chemical properties (reprinted with permission from ref. [78]. © 2024 The Authors. Published by Elsevier Ltd.); (**B**) product recovery solvent systems based on aqueous two-phase extraction in extractive fermentation (reprinted from ref. [83]); (**C**) solubilization and co-solubilization characteristics of pH-responsive Gemini surfactants for polycyclic aromatic hydrocarbons (reprinted with permission from ref. [12]. © 2023 Elsevier Ltd. All rights reserved); (**D**) green solvent selection and reactor optimization strategy in extractive fermentation (reprinted with permission from ref. [79]. © 2024 Wiley-VCH GmbH).

Thirdly, scaling up from laboratory scale to industrial production still faces significant cost challenges, including the synthesis cost of solubilizers, the cost of separation and recovery, and the difficulty in ensuring process reproducibility and stability in complex industrial environments [79]. For instance, in wastewater treatment, when membrane separation is combined with solubilizers, membrane fouling and batch differences in materials may lead to a decline in the reproducibility of flux and retention rate, as shown in Figure 10A [81].

Another prominent point is that the existing solubilization systems usually lack the capability to exhibit an intelligent response to environmental changes and are difficult to self-regulate according to the dynamic changes in pH, temperature and REDOX potential, thereby limiting their application adaptability in complex environments (see Figure 10B) [82].

Finally, the existing solubilization systems have limited treatment capacity for insoluble pollutants, especially when the pollutant concentration is high or its nature is complex. The solubilization efficiency of polycyclic aromatic hydrocarbons is significantly affected by surfactant molecular structure, micellar CMC, hydrophobicity and other related parameters. A single solubilization strategy often fails to balance high efficiency and high stability. Improving solubilization efficiency may come at the cost of sacrificing stability or increasing toxicity. Selective solubilization still presents a major difficulty in the separation of multicomponent mixtures. In addition, the solubilization effect is closely related to the structure, polarity and hydrophobicity of the pollutants. For some extremely hydrophobic or pollutants with special functional groups, the effect of the existing solubilization systems is often poor, as presented in Figure 10C [12,83].

### 4.2. Future Directions

To overcome the above challenges, solubility-based technologies are developing in the following directions, aiming to enhance efficiency, reduce environmental risks and achieve sustainability [77,78,84].

**(1)**  **Intelligent responsive systems**

Future studies should commit to building a “switch-type” intelligent solubilization carrier that has a sensitive response to environmental stimuli such as pH, temperature, and REDOX signals [82,85]. For instance, researchers could develop amphiphilic polymer micelles that can trigger hydrophobic nucleus contraction at specific pH values to enrich pollutants, or carriers that dissociate under REDOX signals to release pollutants [12]. This will significantly enhance the precise controllability of the process, such as achieving green and sustainable phenol production under mild conditions by using low-eutectic solvents in decarboxylation reactions [82].

**(2)**  **Multi-strategy synergy for efficiency**

Studies should explore synergistic strategies, e.g., cyclodextrin-modified polymer micelles and polymer–surfactant composite systems, integrate the advantages of different solubilization mechanisms, and strive to achieve performance breakthroughs [86,87,88]. For instance, cyclodextrin can enhance the solubility of poorly soluble substances through host–guest inclusion. Combining cyclodextrin with polymer micelles can simultaneously utilize the inclusion ability of cyclodextrin and the solubilization ability of micelles, thereby significantly enhancing the solubilization efficiency and selectivity [85]. In addition, the co-solvation strategy is also regarded as a sustainable approach to enhancing the solubility of poorly soluble drugs. The concept involves dissolving multiple substances in a concentrated solution to increase solubility, as shown in Figure 10D [86,89].

Studies should explore synergistic strategies, e.g., cyclodextrin-modified polymer micelles and polymer–surfactant composite systems, integrate the advantages of different solubilization mechanisms, and strive to achieve performance breakthroughs [86,87,88]. For instance, cyclodextrin can enhance the solubility of poorly soluble substances through host–guest inclusion. Combining cyclodextrin with polymer micelles can simultaneously utilize the inclusion ability of cyclodextrin and the solubilization ability of micelles, thereby significantly enhancing the solubilization efficiency and selectivity [85]. In addition, the co-solvation strategy is also regarded as a sustainable approach to enhancing the solubility of poorly soluble drugs. The concept involves dissolving multiple substances in a concentrated solution to increase solubility, as shown in Figure 10D [86,89].

**(3)**  **Computational simulation-driven design**

Molecular simulation tools, including molecular dynamics modeling and quantitative structure–activity relationship analysis, can be employed at a deeper level. These computational approaches help guide the molecular design of solubilizers and support materials with high efficiency and low environmental risk. Through simulation, the interaction mechanism between solubilizers and pollutants, the micelle formation process and stability can be predicted, thereby screening out the most promising candidate materials before the experiment. For instance, molecular dynamics simulations and the COSMO-SAC method have been utilized to improve the extraction performance of 1,3-propanediol by ionic liquids [90].

**(4)**  **Green and sustainability enhancement**

Studies should aim to promote the applied basic research on green alternative materials, e.g., ionic liquids (ILs), DESs, and bio-based surfactants, to reduce the environmental footprint of the technology [8,77,80,84,91,92,93,94].

- Owing to their low volatility, non-flammability, high thermal stability and structural tunability, ionic liquids (ILs) are considered promising replacements for conventional organic solvents [84,95,96]. They are widely applied in environmental remediation scenarios, including wastewater treatment and gas purification. Microextraction of pollutants using ionic liquids is a green technology in water treatment [97]. Ionic liquids can also serve as solvents or catalysts in catalytic polymerization reactions.
- DESs are a new type of green solvent, featuring low toxicity, biodegradability, low cost and tunability. They have attracted much attention in fields such as natural product extraction, pollutant removal and water treatment [82,84,85,91,92,94,98,99,100,101,102]. The preparation of DESs is simple, and its melting point is much lower than that of its components. Through component design, the properties can be highly regulated. For instance, choline chloride–urea (ChCl–urea) type DESs have been utilized in the decarboxylation reaction of hydroxybenzoic acid to produce phenol in a green manner. Hydrophobic DESs have been developed to effectively remove heavy metal ions from aqueous solutions. DESs can also serve as solvents in the ultrasound-assisted extraction of coumarin. The use of low-melting mixture solvents (LoMMSs) as alternatives to DESs has been suggested, so as to further expand the application scope of green solvents [103].
- Microbial and other biosurfactants are characterized by environmental compatibility, low toxicity, and excellent biodegradability, serving as green substitutes for conventional synthetic surfactants. They have been broadly applied in areas including agricultural production and environmental remediation [8].
- In addition, supercritical carbon dioxide (scCO_2_) is an important green solvent with unique physicochemical properties and is widely used in fields such as extraction and material design, for instance, in the design of molecularly imprinted polymers [104]. Combining scCO_2_ with ionic liquids can enhance the efficiency of microextraction of pollutants [97].

Although solubility-based technologies encounter challenges pertaining to solubilization efficiency, toxicity, stability, and cost across environmental the remediation, green chemistry, and chemical separation domains, promising progress is foreseeable. Through the development of intelligent responsive systems, multi-strategy synergies, computational simulation-guided design, and green sustainable solvents, breakthroughs in and extensive applications of these technologies can be anticipated.

## 5. Conclusions

This review elaborates on the strategies, molecular mechanisms, and applications of solubilization of nonpolar substances in polar solvents. Focusing on three major fields, namely environmental remediation, green chemistry, and chemical separation, it discusses four core strategies: surface and interface engineering, host–guest inclusion, solvent engineering, and nanostructured encapsulation. It reveals the influences of driving forces such as hydrophobic effect, van der Waals force, π-π stacking, host–guest interaction, hydrogen bonds, and electrostatic interaction on the solubilization process at the molecular level, and analyzes thermodynamic and kinetic mechanisms such as micelle self-assembly, distribution equilibrium, and mass transfer kinetics.

Although solubilization technology has made significant progress, it still faces many challenges: the environmental toxicity of traditional solubilizers, the insufficient structural stability of micelles and microemulsions under extreme conditions, the high cost of scale-up from laboratory to industrial production, the lack of intelligent response capabilities of existing systems to environmental changes such as pH, temperature, and redox potential, and the limited treatment capacity for high-concentration or complex-property pollutants. In addition, the selective solubilization of multicomponent mixtures remains a major challenge in the field of separation.

Future research can focus on the following directions: (1) developing “switch-type” intelligent solubilization carriers responsive to pH, temperature, light, redox, etc., to achieve controllable cyclic solubilization-release processes; (2) exploring multi-strategy synergistic enhancement schemes such as cyclodextrin-modified polymer micelles and polymer–surfactant composite systems; (3) using computational tools such as molecular dynamics simulation, COSMO-RS theory, and quantitative structure–activity relationships to guide the molecular design of solubilizers and supporting materials; (4) vigorously developing green alternative materials such as ionic liquids, low-melting-point solvents, and biobased surfactants to reduce the environmental footprint of the technology. Employing these approaches is expected to promote the continuous development of solubilization technology towards efficiency, intelligence, and greenness, and achieve greater breakthroughs in practical industrial applications.

## Data Availability

No new data were created or analyzed in this study.
